# Role of the Specialized Proresolving Mediator Resolvin D1 in Systemic Lupus Erythematosus: Preliminary Results

**DOI:** 10.1155/2018/5264195

**Published:** 2018-10-21

**Authors:** Luca Navarini, Tiziana Bisogno, Domenico Paolo Emanuele Margiotta, Alessandra Piccoli, Silvia Angeletti, Alice Laudisio, Massimo Ciccozzi, Antonella Afeltra, Mauro Maccarrone

**Affiliations:** ^1^Unit of Allergology, Immunology and Rheumatology, Department of Medicine, Università Campus Bio-Medico di Roma, Via Álvaro del Portillo 21, 00128 Rome, Italy; ^2^Endocannabinoid Research Group, Institute of Biomolecular Chemistry, Consiglio Nazionale delle Ricerche, 80078 Pozzuoli, Italy; ^3^Unit of Biochemistry and Molecular Biology, Department of Medicine, Università Campus Bio-Medico di Roma, Via Álvaro del Portillo 21, 00128 Rome, Italy; ^4^Unit of Clinical Laboratory Science, Department of Medicine, Università Campus Bio-Medico di Roma, Via Álvaro del Portillo 21, 00128 Rome, Italy; ^5^Unit of Geriatrics, Department of Medicine, Università Campus Bio-Medico di Roma, Via Álvaro del Portillo 21, 00128 Rome, Italy; ^6^Laboratory of Lipid Neurochemistry, European Center for Brain Research, Santa Lucia Foundation, Via del Fosso di Fiorano 64, 00143 Rome, Italy

## Abstract

**Objective:**

Systemic lupus erythematosus (SLE) is an autoimmune systemic disease and its pathogenesis has not yet been completely clarified. Patients with SLE show a deranged lipid metabolism, which can contribute to the immunopathogenesis of the disease and to the accelerated atherosclerosis. Resolvin D1 (RvD1), a product of the metabolism of the omega-3 polyunsaturated fatty acid docosahexaenoic acid (DHA), acts as a specialized proresolving mediator which can contribute in restoring the homeostasis in inflamed tissues. The aim of the present pilot study is to evaluate plasma levels of RvD1 in patients with SLE and healthy subjects, investigating its potential role as a biomarker of SLE and assessing its relationship with disease activity and laboratory parameters.

**Methods:**

Thirty patients with SLE and thirty age- and sex-matched healthy subjects (HSs) have been consecutively recruited at Campus Bio-Medico University Hospital. RvD1 plasma levels were measured by ELISA according to the manufacturer's protocol (Cayman Chemical Co.). RvD1 levels were compared using Mann–Whitney test. Discriminatory ability for SLE has been evaluated by the area under the ROC curve.

**Results:**

Lower levels of RvD1, 45.6 (35.5–57.4) pg/ml, in patients with SLE have been found compared to HSs, 65.1 (39.43–87.95) pg/ml (*p* = 0.0043). The area under the ROC curve (AUC) for RvD1 was 0.71 (95% CI: 0.578–0.82) and the threshold value of RvD1 for the classification of SLE was <58.4 pg/ml, sensitivity 80% (95% CI: 61.4–92.3), and specificity 63.3% (95% CI: 43.9–80.1), likelihood ratio 2.2 (95% CI: 1.3–3.6).

**Conclusions:**

The present preliminary study allows hypothesizing a dysregulation of RvD1 in patients with SLE, confirming the emerging role of bioactive lipids in this disease.

## 1. Introduction

Systemic lupus erythematosus is an autoimmune systemic disease which can involve virtually every organ or apparatus [1]. Despite intense research efforts, the pathogenesis of SLE is not completely understood [2] and many unmet needs still remain in the diagnosis, management, and prognosis [3]. In SLE patients, accelerated atherosclerosis and increased risk of cardiovascular disease (CVD) have been observed [4, 5]. SLE patients are also characterized by an altered lipid metabolism [6], with increased triglycerides, total cholesterol, low-density lipoprotein (LDL) cholesterol, and apolipoprotein B (ApoB) levels, as well as reduced high-density lipoprotein (HDL) cholesterol levels [6]. Oxidation of LDL (oxLDL), which occurs in the early stages of atherosclerosis and can induce inflammation and formation of anti-oxLDL autoantibodies [7], is increased in patients with SLE, and this phenomenon is associated with CVD parameters and renal involvement [5, 8, 9]. Notably, omega-6 polyunsaturated fatty acid (FA) elevations have been observed in SLE [10], and recently, altered metabolism of the endocannabinoid 2-arachidonoylglycerol (2-AG) has been demonstrated in patients with SLE, who show higher plasma levels of this molecule compared to healthy subjects [11]. On the other hand, the role of omega-3 polyunsaturated FAs still remains elusive. Of note, the dietary supplementation of the omega-3 polyunsaturated FAs eicosapentaenoic acid (EPA) and docosahexaenoic acid (DHA) provides improvement of SLE manifestations in 5 of 7 studies [12–16], while one study failed to demonstrate beneficial effects [17] and another one showed an initial improvement followed by loss of effectiveness [18] (see [19] for a recent review).

Omega-3 polyunsaturated FA can be metabolized in inflamed tissue leading to specialized proresolving mediators (SPMs), which prevent further polymorphonuclear cell (PMN) infiltration, induce efferocytosis of apoptotic bodies in macrophages, and promote tissue repair and healing [20–22]. D-series resolvins (RvDs) are derived from DHA and include RvD1-RvD6, while E-series resolvins (RvEs) are derived from EPA and include RvE1-RvE3 [20, 23]. In vivo, the biosynthesis of RvDs, including RvD1, requires a first step involving 15-lipoxygenase (15-LOX) or aspirin-triggered cyclooxygenase-2 (COX-2) and a second step involving 5-LOX [24, 25]. RvD1 exerts its biological functions through interaction with G-protein-coupled receptor 32 (GPR32) or lipoxin A4 receptor/formyl peptide receptor 2 (ALX/FPR2). In addition to the effects on innate immunity, RvD1 is able to support the humoral response that increases IgM and IgG production [26], also reducing IgE secretion [27]. Overall, the output of proinflammatory cytokines from T helper (Th) and cytotoxic lymphocytes and the differentiation of Th1 and Th17 are reduced, and the differentiation of T regulatory (Treg) cells is enhanced [28]. As yet, little is known about the role of RvD1 in rheumatic diseases. For instance, RvD1 shows proresolving features and protects the cartilage from injury in a mouse model of arthritis [29]. In vitro, RvD1 also reduces inflammatory mediators and oxidative stress in osteoarthritis [30].

The present pilot study is aimed at evaluating plasma levels of RvD1 in patients with SLE and healthy subjects, investigating its potential role as a biomarker of SLE and assessing its relationship with disease activity and laboratory parameters.

## 2. Materials and Methods

### 2.1. Study Population and Clinical Assessment

Thirty patients with SLE, classified according to the 2012 Systemic Lupus International Collaborating Clinics (SLICC) criteria [31], were consecutively enrolled from outpatient lupus clinic of the Campus Bio-Medico Università Hospital of Rome. As for the control group, thirty age- and sex-matched healthy subjects (HSs) without chronic diseases and not taking any medication were also enrolled using a “friend of the same age” referral strategy. The study was conducted in compliance with International Conference on Harmonization Good Clinical Practice guidelines and the Declaration of Helsinki. In the SLE cohort, the inclusion criteria included serological disease activity: anti double-strand DNA (anti-dsDNA) positivity and/or low plasma of complement component 3 (C3) and/or C4 with or without extractable nuclear antigen antibodies (anti-ENA), anti-phospholipids, and hypergammaglobulinemia [32]. At enrollment, SLE treatment with low-medium dose glucocorticoids (prednisone <25 mg/day), immunosuppressants (such as methotrexate, cyclosporine, azathioprine, and mycophenolate mofetil), was not an exclusion criterion. In both SLE and HS cohorts, the exclusion criteria included past or present biological therapy (such as rituximab, tocilizumab, or belimumab), glucocorticoids bolus in the previous year, cyclophosphamide treatment in the previous year, cancer at enrollment or in the previous 5 years, infectious diseases at enrollment or in the previous 2 months, and current pregnancy. At enrollment, antinuclear antibodies (ANA), anti-dsDNA, erythrosedimentation rate (ESR), and C-reactive protein (CRP) C3 and C4 levels have been assessed with conventional laboratory tests. In the SLE cohort, disease activity has been measured with the Safety of Estrogens in Lupus Erythematosus National Assessment-SLE Disease Activity Index (SELENA-SLEDAI) and the British Isles Lupus Activity Group (BILAG), while organ damage has been evaluated with the SLICC/American College of Rheumatology (ACR) Damage Index (SDI). At enrollment, a blood sample has been taken and plasma was separated.

### 2.2. Quantification of RvD1

RvD1 plasma levels were measured by ELISA according to the manufacturer's protocol (Cayman Chemical Co., Ann Arbor, MI), as validated elsewhere [33].

### 2.3. Statistical Analysis

Data were expressed as median (25th–75th percentile). RvD1 levels between patients with SLE and HSs as well as demographic and laboratory parameters have been compared using Mann–Whitney *U* test. Fisher's exact test has been used to analyze contingency tables. Receiver operating characteristic (ROC) analysis was used to define the ability of RvD1 to differentiate patients with SLE and HSs; the optimum cutoff value has been identified from the highest Youden's index. Pretest odds, posttest odds, and posttest probability were calculated. Statistical analysis was performed using GraphPad Prism 7 (GraphPad Software, Inc., San Diego, Ca, USA) and MedCalc 11.6.1.0 (Belgium).

## 3. Results

Demographic features and clinical and laboratory characteristics of the patients with SLE and HSs are shown in Table 1.

**Figure 1:**
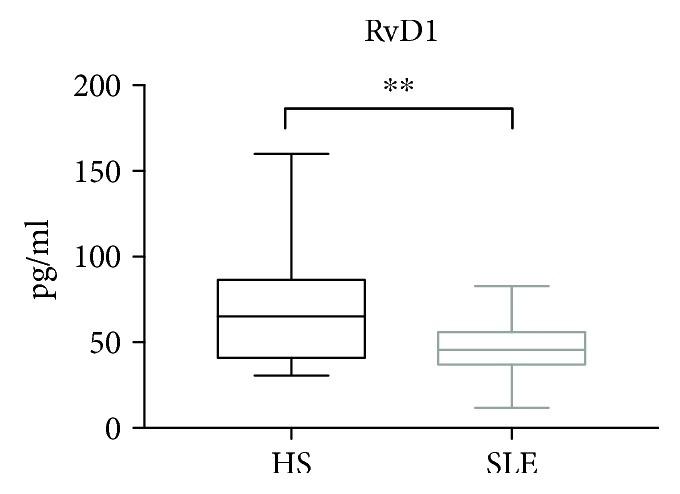
Levels of resolvin D1 (RvD1) in plasma from SLE patients (*n* = 30) and matched HSs (*n* = 30). Data are expressed as pg/ml, median (horizontal bar) with 25th and 75th percentile (boxes), and minimum and maximum (bars) (^∗∗^*p* = 0.0043).

Patients with SLE showed lower levels of RvD1, 45.6 (35.5–57.4) pg/ml, compared to HSs, 65.1 (39.43–87.95) pg/mL (*p* = 0.0043), as reported in Figure 1. Notably, in the SLE cohort, no difference in RvD1 levels has been demonstrated between patients taking low-dose aspirin or not (*p* = 0.11).

**Figure 2:**
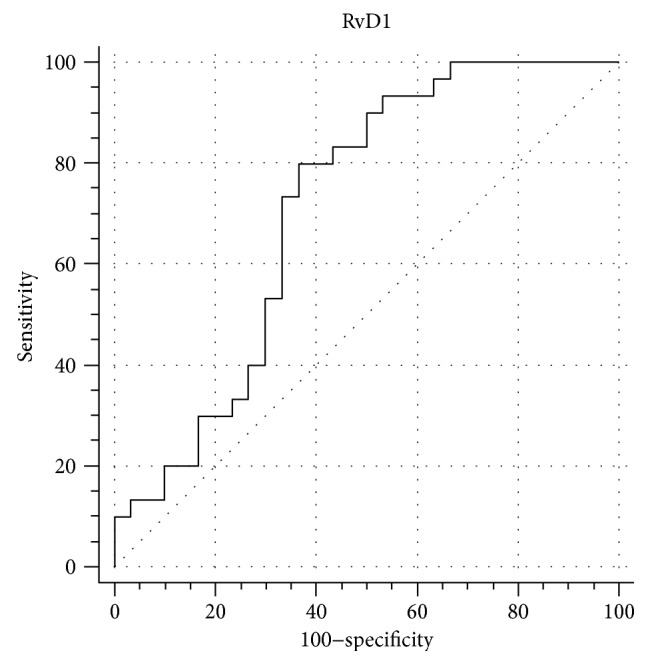
ROC curve for RvD1. Area under the curve (AUC) value is 0.71 (95% CI: 0.58 to 0.82).

The area under the ROC curve (AUC) was 0.71 (95% CI: 0.578–0.82), showing a fair discriminatory ability as reported in Figure 2. Based on ROC and AUC analysis, the threshold value of RvD1 for the classification of SLE was <58.4 pg/ml, sensitivity 80% (95% CI: 61.4–92.3), and specificity 63.3% (95% CI: 43.9–80.1), and likelihood ratio 2.2 (95% CI: 1.3–3.6). Based on the cutoff value of 58.4 pg/ml, the odds ratio of SLE was 0.1522 (95% CI: 0.0488–0.4742), *p* = 0.0012.

**Figure 3:**
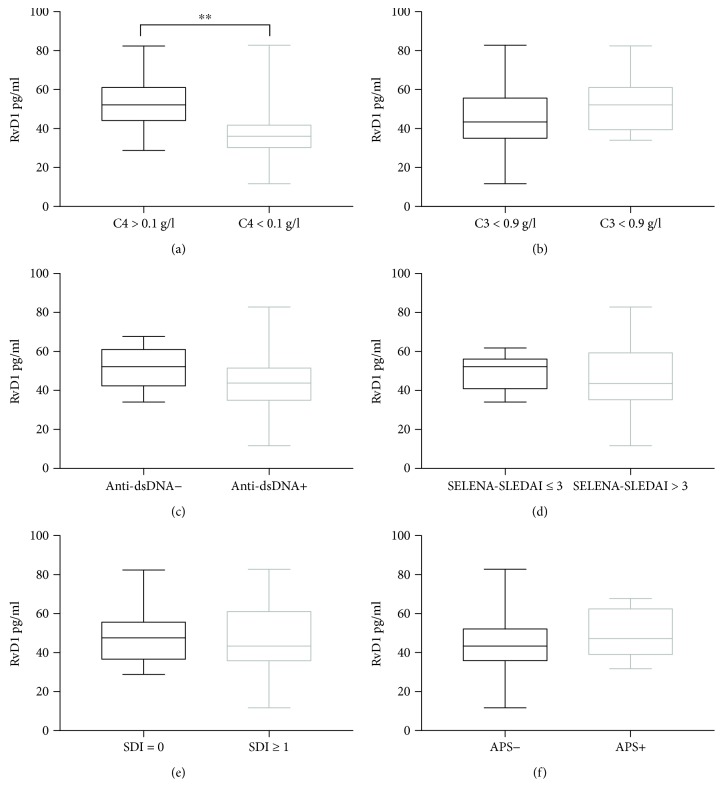
(a) Levels of resolvin D1 (RvD1) in plasma from SLE patients with low C4 levels (<0.1 g/l) (*N* = 10) and SLE patients with normal C4 levels (>0.1 g/l) (*N* = 20). (b) Levels of resolvin D1 (RvD1) in plasma from SLE patients with low C3 levels (<0.9 g/l) (*N* = 19) and SLE patients with normal C4 levels (>0.9 g/l) (*N* = 11). (c) Levels of resolvin D1 (RvD1) in plasma from anti-dsDNA antibody-positive SLE patients (*N* = 18) and anti-dsDNA antibody-negative SLE patients (*N* = 12). (d) Levels of resolvin D1 (RvD1) in plasma from SLE patients with SELENA-SLEDAI ≤ 3 (*N* = 10) and SLE patients with SELENA-SLEDAI > 3 (*N* = 20). (e) Levels of resolvin D1 (RvD1) in plasma from SLE patients with SDI = 0 (*N* = 15) and SLE patients with SDI ≥ 1 (*N* = 15). (f) Levels of resolvin D1 (RvD1) in plasma from SLE patients without antiphospholipid syndrome (APS) (*N* = 21) and SLE patients with APS (*N* = 9). Data are expressed as pg/ml, median (horizontal bar) with 25th and 75th percentile (boxes), and minimum and maximum (bars) (^∗∗^*p* = 0.0087).

In the SLE cohort, no relation has been found between RvD1 plasma levels and disease activity scores, SDI, or disease duration. Likewise, no relation has been demonstrated between RvD1 plasma levels and pharmacological therapies. Nevertheless, SLE patients with low C4 levels (<0.1 g/l) also had lower RvD1 plasma levels, 36.05 pg/ml (29.55–42.45), compared to patients with normal (>0.1 g/l) C4 levels of 52.2 pg/ml (43.4–61.8), *p* = 0.0087, as reported in Figure 3. In the SLE cohort, no significant difference in RvD1 plasma levels has been found among patients with low (<0.9 g/l) and normal (>0.9 g/l) C3 levels.

## 4. Discussion

SLE is a complex autoimmune multisystemic disease, with a huge impact on quality of life and development of organ damage [34, 35]. Despite having a large number of studies clarify many aspects of the pathogenesis of this disease, a comprehensive understanding of the immunological phenomena underlying the clinical manifestations of SLE still remains a challenge [36, 37].

Bioactive lipids seem to be a main actor in inflammation, and they could play a pivotal role in immunopathogenesis on many inflammatory diseases [22, 38, 39]. At present, despite SPMs representing key mediators in rheumatic diseases, data are still scarce and their potential from a therapeutic point of view has not yet been adequately addressed [22].

For the first time, our study demonstrates lower levels of RvD1 in plasma of SLE patients compared to HSs. Moreover, the analysis of the ROC curve showed a fair ability of RvD1 to discriminate SLE patients from HSs, providing a preliminary cutoff value of 58.4 pg/ml. Recently, Barden and coworkers demonstrated higher plasma levels of RvD1 in patients with arthritis compared to healthy subjects; therefore, the role of RvD1 in SLE and arthritis appears to be different [40]. In our cohort, no relation between RvD1 plasma levels and disease activity has been found. However, we demonstrated lower levels of RvD1 in patients with low plasma levels of C4 (but not of C3). This finding supports the hypothesis that RvD1 could affect complement cascade activation, which is a well-established pathogenetic feature of SLE [41].

Several weaknesses of this study should be considered. The sample size was relatively small, and further studies on a larger number of patients are required to better evaluate the role of RvD1 in SLE, especially its potential role as a biomarker. Furthermore, this study did not schedule a follow-up of patients and therefore could not ascertain the role RvD1 in predicting changes in disease activity, damage accrual, or laboratory parameters over time. Moreover, no information about omega-3 polyunsaturated FAs was available. In the present study, we cannot exclude that the difference of RvD1 plasma concentrations between patients with SLE and HSs may partly reflect the use of glucocorticoids or immunosuppressants.

In conclusion, this study allows hypothesizing a dysregulation of RvD1 in SLE and confirms the emerging role of bioactive lipids in this disease.

## Figures and Tables

**Table 1:** Patients' characteristics at enrollment.

	SLE (*n* = 30)	HSs (*n* = 30)	*p*
Age (years)	39 (35–46.25)	40.5 (35–46.5)	ns
Sex (F/M)	29/1	29/1	ns
Disease duration (months)	64 (31–99)	NA	
Antiphospholipid syndrome (*N*)	9	0	0.0019
Anti-dsDNA positivity (*N*)	18	0	<0.0001
Hypcomplementemia C3 (*N*)	19	0	<0.0001
C3 (g/l)	0.69 (0.34–0.8)	1.01 (0.96–1.2)	0.0002
Hypocomplementemia C4 (*N*)	10	0	0.0008
C4 (g/l)	0.078 (0.03–0.115)	0.15 (0.1–0.225)	0.0039
No prednisone (*N*)	4	30	<0.0001
Prednisone ≤5 mg (*N*)	13	0	0.0003
Prednisone >5 mg	13	0	0.0003
Immunosuppressants (*N*)	18	NA	
Hydroxychloroquine (*N*)	19	NA	
Low dose aspirin (*N*)	8	0	0.0046
SELENA-SLEDAI	4 (2–6.75)	NA	
BILAG A	4	NA	
BILAG B	9	NA	
SDI	0.917 (0–1.04)	NA	

NA: not applicable; ns: not significant.

## Data Availability

The data used to support the findings of this study are available from the corresponding author upon request.
